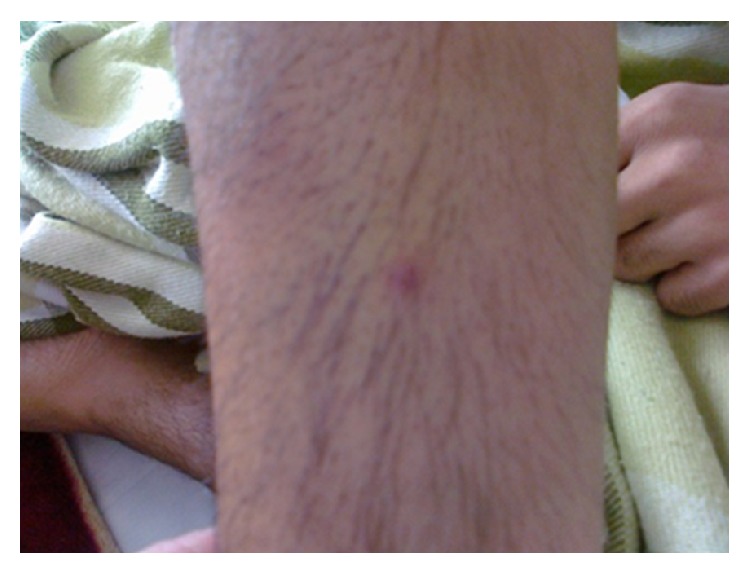# Pulmonary Nodules as an Initial Manifestation of Behçet's Disease

**DOI:** 10.1155/2014/869817

**Published:** 2014-11-09

**Authors:** M. Malekmohammad, A. Emamifar

**Affiliations:** National Research Institute for Tuberculosis and Lung Disease (NRITLD), Masih Daneshvari Hospital, Niavaran, P.O. Box 19575, Darabad, Tehran 1955841452, Iran

## Abstract

Behçet's disease (BD) is a systemic vasculopathy, characterized by recurrent oral aphthae, genital ulcers, uveitis, and skin lesions. Although vascular involvement, including venous and arteries of any size, is a usual manifestation, cases with pulmonary thrombosis as the initial symptom are not common in the absence of pulmonary artery aneurysm (PAA). This report describes a 36-year-old man with recurrent fever, nonmassive hemoptysis, and persistent cough with lung nodules in CT scan who had undergone open lung biopsy. On the basis of morphological findings, BD was suggested and more precise evaluation confirmed the diagnosis.

## 1. Introduction

Behçet's disease is a chronic, inflammatory disorder of unknown etiology which is described by Turkish dermatologist Hulusi Behçet [1, 2]. Recurrent oral aphthae, which are essential sign, genital ulcers, and ocular inflammation are the cornerstones of Behçet's manifestation [1, 3]. It affects mainly young adults, M/F = 2–5/1, with a geographical distribution from the Mediterranean countries to far east [2, 3]. International study group published a set of diagnostic criteria for Behçet's disease which require the presence of oral ulceration and any other involvement of two organs including recurrent genital ulceration, eye lesions, skin lesions, or positive pathergy test [2]. We report a case of Behçet's disease pulmonary thrombosis as the initial symptom.

## 2. Case Report

A 32-year-old man was referred to Masih Daneshvari Hospital with unexplained fever, cough, hemoptysis, and weight loss. These complications had begun 2 months before. Following two periods of hospitalization and medical treatment without any improvement, the patient was admitted here with a CT scan showing only right subpleural nodules.

**Figure 1:**
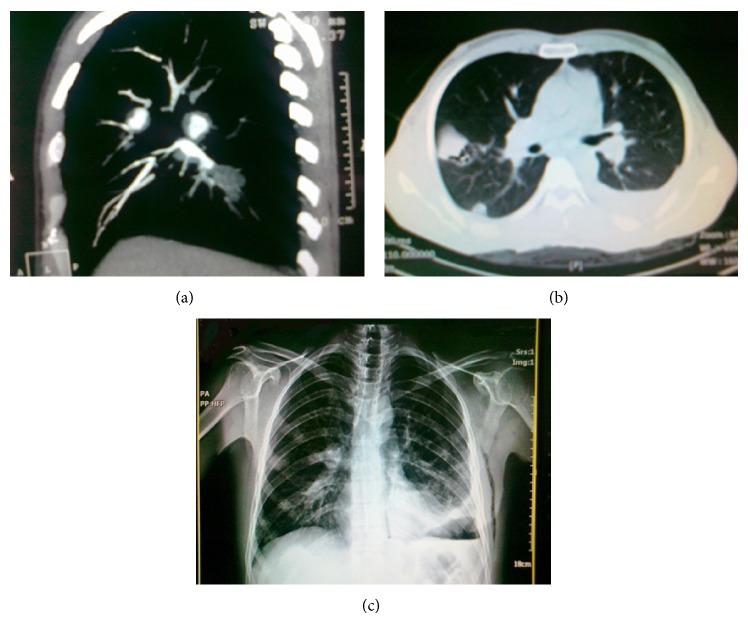


On admission, the patient appeared cachectic. His blood pressure was 110/70 mmHg, pulse and respiratory rates were 112/min and 22/min, respectively, and oral temperature was 38.3. Inspiratory crackles were heard at both lung bases. Both legs' skin nodular lesions were the other findings. The erythrocyte sedimentation rate (ESR) was 125 mm/h and D-Dimer was 2577 *μ*g/L. Other hematologic, biochemistry, and serologic tests ANA and ANCA were within normal limit. Imaging studies of the thorax revealed multiple diffused bilateral pulmonary nodules, left lingual consolidation, and pleural effusion (Figure 1). Open lung biopsy of lingual lesion had been done. Microscopic examination showed areas of hemorrhage and organized thrombi and foci of vascular proliferation. We suspected lung involvement by BD and commented for more clinical evaluation. In a rheumatology consult, a detailed past medical history revealed that he had been involved with recurrent oral and genital aphthous. Additionally, he had episodes of painful tender red nodules over the legs suggestive of erythema nodosum (EN). He did not use any drugs for these complaints. In the internal ward, we considered that, within 48 hours of insertion of intravenous cannula as well as venipunctures, pustular vesicles appeared at puncture sites (a positive pathergy test) (Figure 2). Based on these findings, a clinical diagnosis of Behçet's disease was made. 1000 mg intravenous pulse methylprednisolone for three days started and partial remission was achieved.

## 3. Discussion

Behçet's disease (BD) is a multisystem disorder characterized by vasculitis, first described in 1937 [4]. The diagnostic criteria are oral ulceration and any two of genital ulceration, eye lesions (most commonly uveitis), pustular skin lesions, and a positive pathergy test [5]. Eyes, skin, joints, the oral cavity, blood vessels, and central nervous system are usually involved, although the heart, lung, kidney, genital system, and gastrointestinal tract may be affected less frequently [6]. It usually shows episode of exacerbation and remission, and the prognosis is good unless vital organs are affected [7]. The pathology of the lesions consists of widespread vasculitis that affects arteries and veins of all sizes [8–10]. Pathologically, there are three important vascular abnormalities: perivascular lymphocyte cuffing, transmural polymorphonuclear infiltration, and spontaneous intravascular thrombosis [11].

Pulmonary involvement occurs in up to 8% of cases, most commonly as multiple pulmonary artery aneurysms (PAA) [12]. Male gender is a risk factor for PAA and this is one of the causes of deaths in BD [10, 13]. Although deep venous thrombosis of the lower extremities frequently accompanies pulmonary artery aneurysms, pulmonary thromboembolism is very rare in Behçet's disease because the thrombi in inflamed veins are strongly adherent [13]. Pathological findings in the lung in a patient with Behçet's disease show lymphocytic and necrotizing vasculitis involving all sized pulmonary arteries, veins, and septal capillaries [8].

Pulmonary manifestations of Behçet's disease are including hemoptysis, dyspnea, cough, sputum, chest pain, and fatigue. These symptoms are similar in microscopic vascular disease, macroscopic vascular disease, and nonvascular pulmonary disease, except for massive hemoptysis which is only seen in patients having PAA. Conventional chest radiography was the most common diagnostic method for initial evaluation of pulmonary involvement. High resolution CT is useful in demonstrating parenchymal lesions due to PAA and infarct and pulmonary artery thrombus. Spiral CT angiography is the best radiological tool for the evaluation of pulmonary problems in BD [14]. Histological examination of biopsies or surgical specimens may be helpful in determining the pathological features of structures affected by Behçet's disease [15].

The distinction of pulmonary vasculitis is important because treatment and prognosis may be different in spite of similarities in clinical signs and symptoms of microscopic and macroscopic pulmonary disease. Another factor that may affect treatment is the type of macroscopic pulmonary vascular disease that is PAA and/or pulmonary artery thrombus [14].

In conclusion, we reported a case of BD with an unusual but life threatening presentation and discussed its importance briefly. Additionally, early diagnosis with appropriate treatment can prevent mortality and morbidity in BD.

## Figures and Tables

**Figure 2:**